# The correlation between myopia severity and stress–strain index (SSI) using the Corneal Visualization Scheimpflug Technology (Corvis ST)

**DOI:** 10.1038/s41598-025-23834-x

**Published:** 2025-11-17

**Authors:** Xiaorui Wang, Dan Wang, Sally Hayes, Siân R. Morgan, Craig Boote, Yuhui Duan, Shengjian Mi, Colm McAlinden

**Affiliations:** 1 Department of Ophthalmology, The First Affiliated Hospital of Xi’an Jiaotong University, Xi’an, Shaanxi China; 2 Structural Biophysics Group, School of Optometry and Vision Sciences, Cardiff University, Cardiff, Wales UK; 3 Department of Ophthalmology, The First Affiliated Hospital of Northwest University, Xi’an, Shaanxi China; 4 Xi’an Gaoxin Hospital, Xi’an, Shaanxi, China; 5 Eye & ENT Hospital of Fudan University, Shanghai, China; 6 School of Optometry and Vision Sciences, Cardiff University, Cardiff, Wales UK

**Keywords:** Corneal biomechanics, Myopia severity, High myopia, Stress–strain index, Corvis ST, Stiffness, Medical research, Risk factors, Signs and symptoms

## Abstract

This study aimed to investigate the correlation between myopia severity and the stress–strain index (SSI), measured with the Corneal Visualization Scheimpflug Technology (Corvis ST) device. The subjects were divided into two groups, based on both the axial length (AL) and spherical equivalent refraction (SER): 22–26.00 AL group (22 mm < AL < 26.00 mm) associated with SER of less than − 6.00D, and ≥ 26.00 AL group (AL ≥ 26.00 mm) associated with SER over − 6.00D. The differences in the Corvis ST-derived dynamic corneal response parameters and stiffness parameters between the two groups were investigated. The correlation between SSI and AL, SER, age, ratio of AL to radius of corneal curvature (CR) (AL/CR), and axial length minus anterior chamber depth (ACD) (AL-ACD) were analyzed. The SSI (0.95 ± 0.13 in the 22–26.00 AL group and 0.86 ± 0.15 in the ≥ 26.00 AL group) were significantly different between the two groups (*P* < 0.01). In the ≥ 26.00 AL group, there was evidence of a weak negative correlation between SSI and AL (r = − 0.265, *P* < 0.01), AL/CR (r = − 0.376, *P* < 0.01), and AL-ACD (r = − 0.224, *P* < 0.01); and a weak positive correlation between SSI and SER (r = 0.251, *P* < 0.01). However, in the 22–26.00 AL group, there was no correlation between SSI and AL, AL-ACD, AL/CR or SER (*P* > 0.05). SSI was significantly correlated with AL, which is the major determinant of SER, in the ≥ 26.00 AL group. This correlation was not affected with CR and ACD, as both AL/CR and AL-ACD also correlated with SSI at the same degree.

**Supplementary Information:**

The online version contains supplementary material available at 10.1038/s41598-025-23834-x.

## Introduction

Myopia currently affects over one-quarter of the global population and this is expected to rise to 50% by 2050. Further, approximately 10% are predicted to be highly myopic (> 6 dioptres (D))^1^^,^^2^. Myopia progression is primarily associated with axial lengthening of the eye, driven by scleral collagen remodelling, and a thinning of the sclera and underlying choroid^3^. The mean sub-foveal posterior scleral thickness in highly myopic populations is thinner than the mean anterior scleral thickness by more than 45% along all meridians^4^. Since the anterior scleral connective tissue is anatomically continuous with the neighbouring cornea and shares a similar collagen-rich structural composition^5^, it may be assumed that altered corneal and scleral biomechanics are intrinsically related^6^. Accordingly, there is a burgeoning interest amongst researchers to measure in vivo corneal biomechanical changes in myopia.

The measurement of corneal biomechanical characteristics is challenging, due to the complex and viscoelastic nature of the tissue^7,8^. A prominent in vivo biomechanical measurement device is the dynamic Scheimpflug imaging analysis system (Corneal Visualization Scheimplug Technology [Corvis ST], Oculus Optikgeräte GmbH, Wetzlar, Germany, software version V.1.6b2507; https://www.oculus.de/en/products/corvis-st/), which uses a standardised air-puff of 60 mmHg^9^. The latest software version of the Corvis ST device offers multiple measurement parameters, including the Ambrósio Relational Thickness to the horizontal profile (ARTh) which provides a measure of the corneal horizontal thickness profile, and dynamic corneal response values which describe the speed, the time point, and the deformation amplitude of the cornea at a certain phase (e.g. the stiffness parameter at the first applanation (SP-A1). It also provides a deflection amplitude ratio (Def A ratio), which describes the ratio between the deflection amplitude at the corneal apex [Def A (apex)] and the deflection amplitude at 2 mm from the apex [Def A (2 mm)]. Unlike the deformation amplitude, which provides a measure of the corneal displacement including whole eye movement, the deflection parameters take into account, and compensate for, the movement of the whole eye. Additionally, it provides a Corvis Biomechanical Index (CBI), that incorporates parameters such as ARTh and SP-A1, and a Stress–Strain Index (SSI), and aims to provide a measure of tissue stiffness that is independent of both intraocular pressure (IOP) and corneal geometry^10^. The SSI algorithm is based on predictions of corneal behaviour generated using finite element numerical modelling of the effects of IOP and the air-puff on the tissue^10^. The eye model is divided into four regions to incorporate the stress–strain behaviours of the cornea, limbus, anterior sclera, and posterior sclera. This renders the SSI index a reasonably indicative stiffness parameter for the whole of the eyeball tunic. Although SSI has been designed to measure tissue stiffness without the effect of central corneal thickness (CCT) and IOP^10^, several clinical studies have found weak correlation with CCT and IOP, or biomechanically corrected IOP (bIOP)^10–12^. However, SSI has proven to be a sensitive parameter in the evaluation of keratoconus diagnosis and progression^13^, and for the assessing corneal stiffness improvement after corneal crosslinking for keratoconus^14,15^, or post corneal laser refractive surgery combined with corneal crosslinking^16^.

The correlation between corneal biomechanical behaviour and myopic status has been investigated by several groups, with results contrasting largely between studies^17–19^. Several potentially confounding factors may underlie these findings. Firstly, a large age range of participants were enrolled across the studies, leading to possible biomechanical variations between adolescent and adult participants. Secondly, values of axial length (AL) reported in isolation may not well reflect axial myopia severity, especially in eyes featuring a flatter cornea^17^^,^^20^. When Liu et al.^18^ sub-grouped participants by AL to reflect the severity of myopia, they found that SSI was lower in the severely elongated (AL ≥ 26 mm) highly myopic eyes than in those with moderately elongated eyes (AL < 26 mm). Moreover, SSI was found to be negatively correlated with AL in the lower myopia group, but not in the high myopia group^18^. Although no correlation was found between SSI and AL, Chu et al.used the ratio of AL to radius of corneal curvature (AL/CR) as an indicator of myopia severity, and found that SSI was negatively correlated with AL/CR in those with and without high myopia^17^. As the cornea is responsible for around 75% of the refractive power of the eye, AL/CR has been proposed as a more reliable variable for quantifying the degree of myopia than AL alone^20^.

The purpose of this study was to investigate whether a correlation exists between the severity of myopia and SSI, derived from corneal stiffness parameters measured by Corvis ST.

## Methods

### Participant details

This prospective study included 318 participants who were consecutively recruited from May 2019 to March 2022 at the First Affiliated Hospital of Xi’an Jiaotong University in China. The study was approved by the Ethics Board of the First Affiliated Hospital of Xi’an Jiaotong University Institute and was performed in compliance with the tenets of the Declaration of Helsinki. Written informed consent was obtained from all participants prior to enrollment in the study. Exclusion criteria included the following conditions: aged < 18-years-old, rigid contact lens use, currently pregnant or nursing, ocular surgery, and systematic diseases, abnormally high or low IOP (> 21 mmHg or < 10 mmHg), keratoconus and other corneal pathologies.

In order to evaluate the correlation between corneal biomechanical parameters and myopia severity, subjects were divided into two groups based on both the AL and SER value: 22–26.00 AL group (22 mm < AL < 26.00 mm) associated with SER of greater than − 6.00D; ≥ 26.00 mm AL group (AL ≥ 26.00 mm) associated with SER of less than -6.00D. Patients with very flat corneas associated with long AL, for example, a case with − 3.00D of SER but associated with 26.34 mm AL, were considered to have a non-proportional eye structure and their data were excluded from the study (n = 60). In total, 258 patients (148 females and 110 males, mean age 30.30 ± 9.76 years, range 18 to 52) met the inclusion criteria and were enrolled. Only data from the right eye was included in the analyses to avoid bias associated with bilateral eye correlation.

All subjects underwent comprehensive ophthalmic examinations, including slit-lamp biomicroscopy, fundoscopy, auto-refraction (AR-1, NIDEK, Japan, software version 1.10.02; https://www.nidek-intl.com/product/ophthalmology/refraction/auto-refractometer/ar-1.html), subjective manifest refraction with an undilated pupil, non-contact tonometry (NT-510, NIDEK, Japan, software version 1.07.00; https://www.nidek-intl.com/items/non-contact-tonometer-nt-530-510-2/), and biometry with the IOL-master 500 (Carl Zeiss Meditec AG, Germany, software version 5.5.0.0062; https://www.zeiss.com/iolmaster), and Scheimpflug corneal tomography with the Pentacam HR (Oculus Optikgeräte GmbH, Wetzlar, Germany, software version 1.26r28; https://www.pentacam.com). The SER was calculated from a dry subjective refraction, undertaken by the same optometrist. Soft contact lens wearers were required to cease contact lens wear for at least 7 days prior to measurements.

### Corneal biometric measurements

The CCT and anterior corneal curvature of the central 3 mm were acquired using the Pentacam HR. AL was measured using the IOL-Master 500. The measurements were repeated five times, with subjects being asked to blink regularly between measurements to ensure tear film stability. The mean value of the AL was used. All measurements were acquired centered on the corneal vertex (normal apex) and performed in a medium-dark room. Only the “OK” quality of image was saved and applied in the data analysis.

### Corneal biomechanical measurements

All Corvis ST measurements were conducted by the same experienced doctor (XRW) in a medium-dark room. If the first capture by the Corvis ST displayed an error such as “Model Deviation”, “Lost Images” or “Alignment”, the patient was tested again until the quality specification button showed the “OK” signal. Although we have previously shown good repeatability from continuous Corvis ST measurements^21^, in this study, 5-min intervals were applied between repeated captures of Corvis ST to eliminate any possible measurement bias. The order of these three tests was IOL-Master, Pentacam and Corvis ST. All the tests were at the time between 10:00–17:00 to avoid the diurnal variations.

### Statistics

Analyses were performed using the statistical software SPSS version 24.0 (IBM, Armonk, New York, USA) and GraphPad Prism Version 9.4.1 (458) (San Diego, USA). Normality of data distributions was confirmed by means of the Kolmogorov–Smirnov test. Demographic data and Corvis ST-derived parameters were continuous data, which were expressed as mean ± standard deviation (SD). An independent-samples t-test was used to compare basic data between the two groups after testing the normal distribution, whereas Pearson’s χ2 test was used for categorical variables. A general linear model analysis of covariance (ANCOVA) was used to adjust for the effect of age, CCT and CR when comparing the difference between the Corvis ST derived corneal biomechanical parameters between the two groups. Pearson correlation analysis was used to assess the correlation between SSI and patients’ demographic data in the two groups separately. Simple linear regression was used to compare SSI and ocular and demographic parameters between the two AL groups. The slope of the two groups was then compared using ANCOVA. Stepwise multivariable linear regression analysis was calculated to study the relationship between SSI and demographic, ocular characteristics (unstandardized β and p value were reported). A *p* value < 0.05 was considered statistically significant. As it is estimated that the correlation coefficient between the SSI and AL in the ≥ 26.00 mm AL group is − 0.4, to achieve significant result (*p* < 0.05) with sufficient power (90%) to detect at least correlation coefficient of 0.3, the minimum required sample size for each group would be 112, based on the algorithm originally described by Guenther^22^.

## Results

### Demographic and biometric data of study participants

Of the 318 participants recruited for the study, 258 met the inclusion criteria described in the Methods. In agreement with the well-established fact that the main cause of myopia is due to lengthening of the axial length, the data from the 258 participants showed a strong correlation between SER and AL (Figure S1).

Data from the 258 participants were divided into two groups according to their AL and severity of myopia, 22–26.00 AL group and ≥ 26.00 AL group; each group contained 113 and 145 cases respectively. The two groups comprised approximately equal numbers of male and female participants but showed significant differences in age, CR, CCT and ACD (all *P* values < 0.05) (Table 1). The two calculated parameters, AL-ACD and AL/CR, also showed significant difference between the two groups (Table 1). The demographic and biomechanical information from the 60 cases excluded from the study for having a non-proportional eye structure, along with the Corvis ST parameters and correlation analysis, are summarised in the supplementary materials (Supplementary Tables S1, S2, S3 and S4).

**Table 1:** Demographic and biometric data.

	22–26.00 AL group Mean ± SD	≥ 26.00 AL group Mean ± SD	*P*
Sex	Female (61.1%)	Female (54.5%)	0.289
	Male (38.9%)	Male (45.5%)	
Age (years)	33.06 ± 10.34	28.14 ± 8.74	< 0.01†
AL (mm)	24.96 ± 0.69	27.41 ± 1.08	< 0.01†
SER (D)	− 4.14 ± 1.22	− 9.45 ± 2.53	< 0.01†
CR (mm)	7.75 ± 0.21	7.82 ± 0.24	0.016*
CCT (μm)	514.13 ± 30.33	527.58 ± 35.84	< 0.01†
ACD (mm)	3.07 ± 0.31	3.22 ± 0.28	< 0.01†
AL-ACD (mm)	21.90 ± 0.70	24.19 ± 1.11	< 0.01†
AL/CR ratio	3.22 ± 0.09	3.51 ± 0.16	< 0.01†

AL, axial length; SER, spherical equivalent refraction; CCT, central corneal thickness; ACD, anterior chamber depth; AL-ACD, axial length minus anterior chamber depth; CR, radius of the corneal curvature value of the anterior surface around a 3 mm ring; AL/CR ratio, axial length divided by radius of the corneal curvature of anterior surface around a 3 mm ring. *P-value < 0.05; †P-value < 0.01.

### Comparison of Corvis ST parameters between groups

Table 2 showed that the Corvis ST measurements of dynamic corneal responses were not significantly different between the 22–26.00 AL and ≥ 26.00 AL groups (*P* > 0.05), apart from the stiffness parameter SSI which was significantly different between the two groups (*P* < 0.01). The ≥ 26.00 AL group showed softer behaviour than the 22–26.00 AL group under the same conditions of bIOP, age and CCT (adjusted CCT was 521.7 μm, adjusted age was 30.3 years and adjusted CR = 7.79), although there was no significant difference.

**Table 2:** Comparisons of Corvis-ST derived dynamic corneal response and stiffness parameters between the normal eye and long eye group.

	22–26.00 AL group (113 OD eyes) Mean ± SD	≥ 26.00 AL group (145 OD eyes) Mean ± SD	*P*
bIOP (mmHg)	14.71 ± 1.85	15.15 ± 1.82	0.227
Def A(2 mm)	0.96 ± 0.09	1.09 ± 0.69	0.053
Def A ratio (2 mm)	6.33 ± 1.00	6.07 ± 0.87	0.843
CBI	0.19 ± 0.29	0.12 ± 0.24	0.185
SP-A1	85.67 ± 14.18	91.27 ± 15.14	0.406
SSI	0.95 ± 0.13	0.86 ± 0.15	< 0.01†

bIOP, biomechanically corrected intraocular pressure; Def A (2 mm), deflection amplitude at 2 mm; Def A ratio (2 mm), ratio between the deflection amplitude at the apex and the deflection amplitude at 2 mm from the apex; CBI, corvis biomechanical index; SP-A1, stiffness parameter at first applanation; SSI, stress–strain index. ANCOVA was used in the comparisons of Corvis-ST derived DCRs and stiffness parameters between the normal eye and long eye groups. A Bonferroni correction was applied in pairwise comparisons after adjusting the effect of CCT, age and CR, adjusted CCT = 521.7 μm, adjusted age = 30.3 years, and adjusted CR = 7.79. †*P* value < 0.01.

### Correlation between SSI and biometric values

**Fig. 1:**
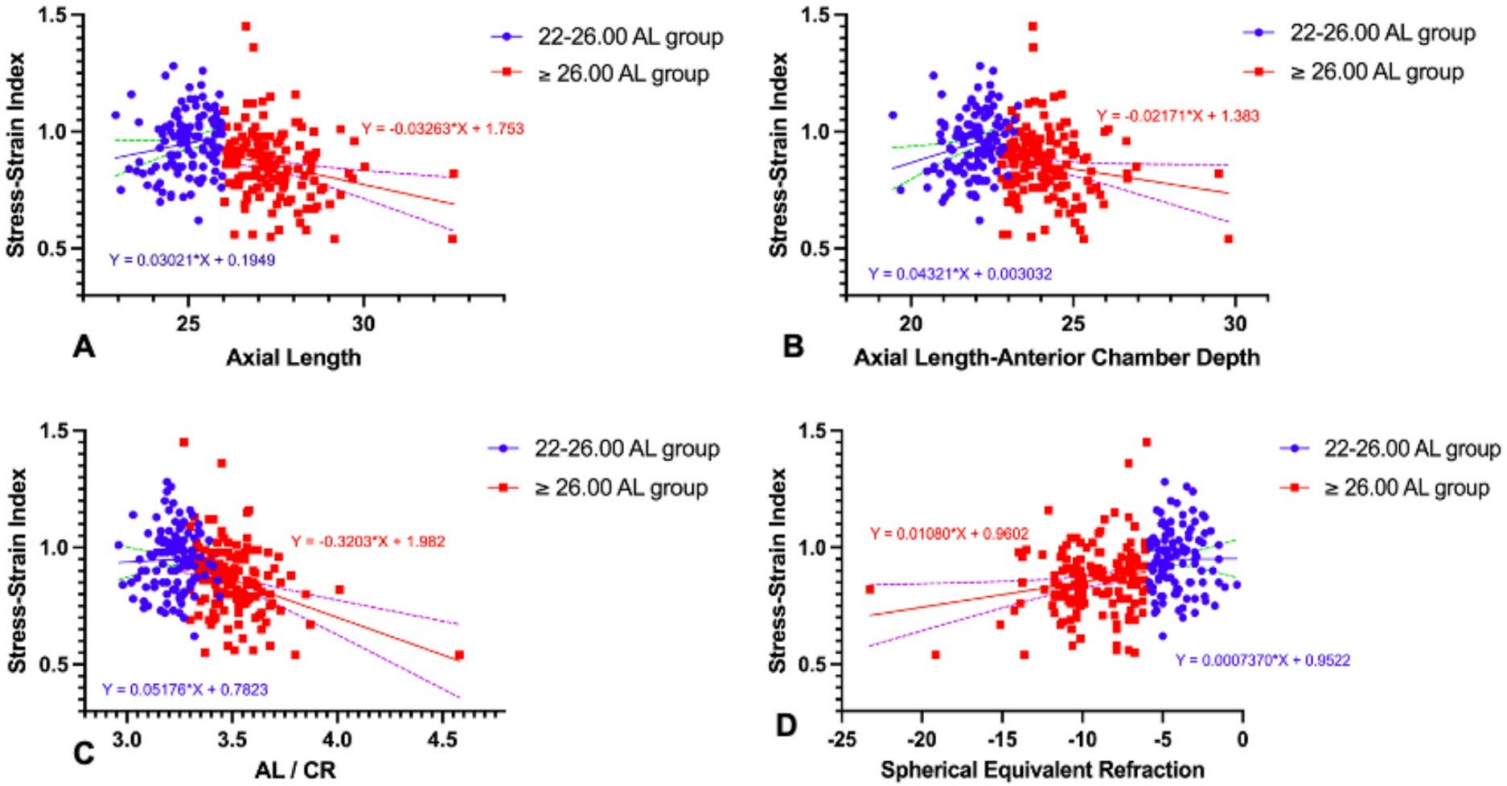
Linear regression between the stress–strain index and axial length (**A**), AL-ACD distance (**B**), AL/CR ratio (**C**), and spherical equivalent refraction (**D**), in both the 22–26.00 AL and ≥ 26.00 AL group. The slopes of the regression lines for the 22–26.00 AL and ≥ 26.00 AL group were significantly different in panels A (*P* = 0.004), B (*P* = 0.003) and C (*P* = 0.017) but there was no significant difference between the regression lines in panel D.

To understand the significance of SSI that is different between the two groups, the correlation between SSI and the biometric values was examined. In the ≥ 26.00 AL group (Table 3), a weak but statistically significant negative correlation was seen between SSI and AL (Fig. 1A), SSI and AL-ACD distance (Fig. 1B), and SSI and the AL/CR ratio (Fig. 1C). There was also a weak positive correlation between SSI and SER (Fig. 1D). In the 22–26.00 AL group, there was no evidence of any correlation between SSI, AL/CR or SER (Table 3; Figs. 1A–D), with the exception of AL, AL-ACD.

**Table 3:** Correlation analysis between SSI and biometric values.

	22–26.00 AL group		≥ 26.00 AL group	
	*r*	*P*	*r*	*P*
SSI vs AL	0.156	0.049*	− 0.242	< 0.01†
SSI vs (AL-ACD)	0.228	< 0.01†	− 0.166	0.023†
SSI vs AL/CR	0.039	0.371	− 0.343	< 0.01†
SSI vs CR	0.119	0.105	0.177	0.017*
SSI vs CCT	− 0.083	0.384	− 0.180	0.030*
SSI vs SER	0.007	0.472	0.189	0.012*
SSI vs age	0.297	< 0.01†	0.235	< 0.01†
SSI vs bIOP	0.374	< 0.01†	0.490	< 0.01†

Pearson correlations were used. *P value < 0.05; †*P* value < 0.01.

**Fig. 2:**
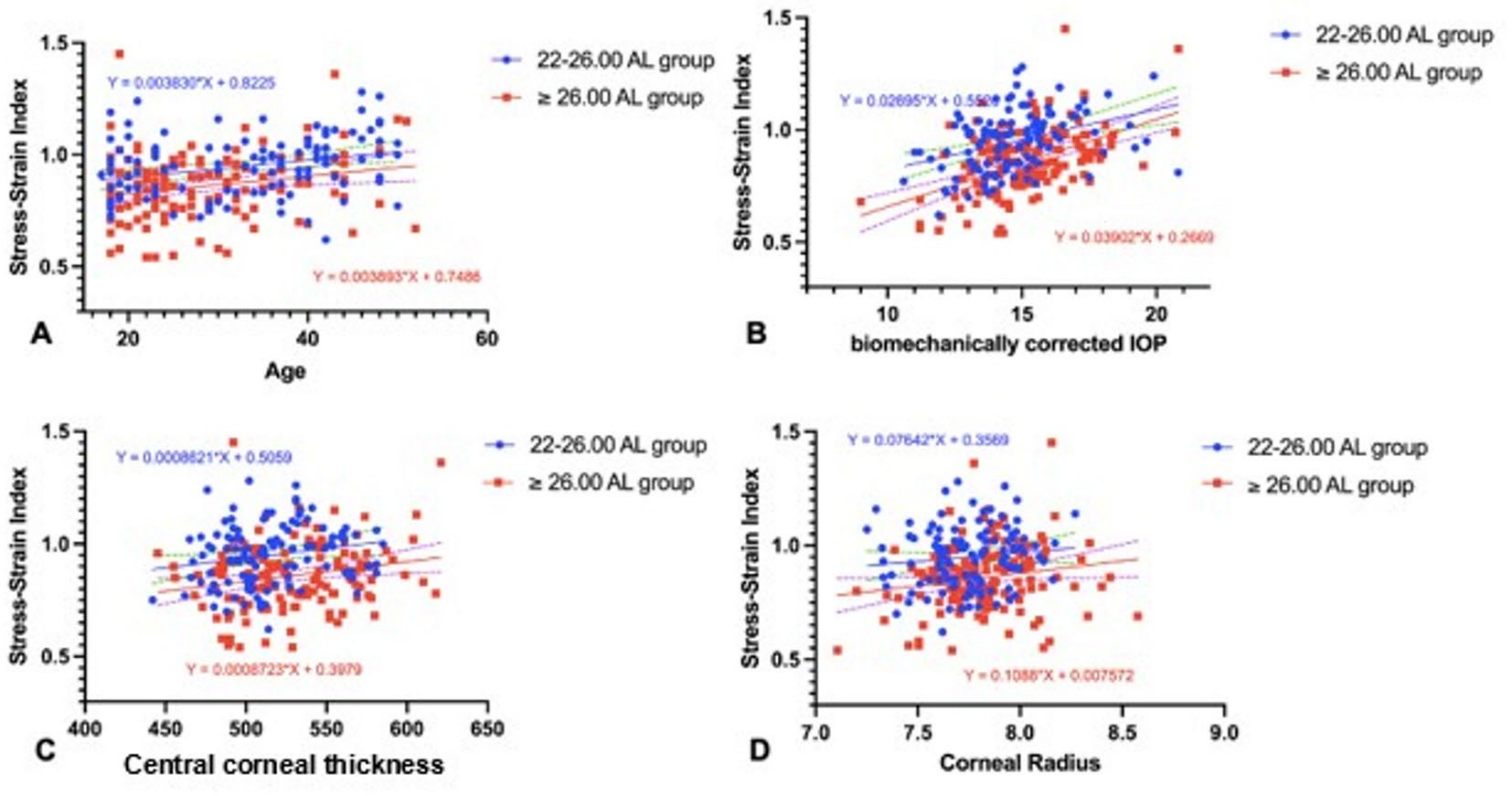
Linear regression between stress–strain index and age (**A**), and biomechanically corrected IOP (**B**), central corneal thickness (CCT) (**C**), and corneal radius (CR) in the 22–26.00 AL and ≥ 26.00 AL group. The slopes of the regression lines for the 22–26.00 AL and ≥ 26.00 AL groups were not significantly different between in all panels.

As age is known to be a significant factor for the stiffness of cornea^10^, the relationship between SSI and age in each of the study groups was examined. In both the 22–26.00 AL and ≥ 26.00 AL groups, SSI was found to be positively correlated with age (r = 0.249, *P* < 0.01 and r = 0.209, *P* < 0.01) (Fig. 2A). Because SSI is intended to be independent of IOP and corneal geometry^10^, analysis was undertaken to determine if there was any evidence of correlation between these parameters in the two groups. The results showed no correlation between SSI and CCT (Fig. 2C) or CR (Fig. 2D). However, there was a correlation between SSI and bIOP in both the 22–26.00 AL and ≥ 26.00 AL groups (r = 0.513, *P* < 0.01 and r = 0.540, *P* < 0.01 respectively) (Fig. 2B).

### SSI association with age, bIOP and biometric values through multiple linear stepwise regression analysis

In order to assess whether these independent variables showing significant correlation with SSI (Table 4) could be used to predict their contribution to SSI, multiple linear stepwise regression analysis was performed. Table 4 described the coefficient (unstandardized β) and P value for multivariate regression analysis.

**Table 4:** Stepwise multivariate regression analysis of SSI and demographic and ocular values.

Parameters	*Age* β (*p*)	*AL* β (*p*)	SER β (*p*)	bIOP β (*p*)	CR β (*p*)	AL/CR β (*p*)	AL-ACD β (*p*)
SSI in 22–26.00 AL group	0.006, (< 0.001)	–	–	0.037, (< 0.01)	0.132, (0.01)	–	–
SSI in ≥ 26.00 AL group	0.004, (< 0.01)	− 0.081, (0.044)	–	0.040, (< 0.01)	–	− 0.374, (< 0.01)	0.095, (< 0.01)

In the 22-26.00 AL group, by stepwise linear regression, equation would be SSI = − 0.819+0.037bIOP+0.006age+0.132CR.

In the ≥ 26.00 AL group, by stepwise linear regression, equation would be SSI = 1.352+0.04bIOP+0.004age− 0.374AL/CR+0.095(AL-ACD) − 0.081AL.

## Discussion

It is well established that myopic progression is associated with choroidal and scleral tissue thinning, and changes in scleral collagen fibril alignment and protein expression^5,23^. However, in recent years, emerging evidence points to the contribution of the cornea in the ocular biomechanical changes that underpin myopia. In particular, avian myopia models and related retinopathies characterised by globe enlargement indicate significant remodelling of the corneal extracelluar matrix^24,25^, with accompanying alterations in tissue elastic modulus and related proteomic changes^26–29^.

*In-vivo* corneal biomechanical measurements in human subjects first became feasible with the development of the Ocular Response Analyzer (ORA; Reichert, Inc., Depew, NY, USA) which utilises infrared light technology to measure the biomechanical response of the eye to a rapid air-jet induced deformation of the cornea^7^. Another newly developed system is the Corvis ST which uses an air stream to deform the cornea inwards and high-speed Scheimpflug imaging to record both the deformation and recovery of the cornea. Various studies have shown good repeatability of biomechanical measurements using the Corvis ST system^30,31,32^, although the repeatability of measurements was found to be slightly better in corneas with a normal or greater central corneal thickness compared to thin corneas measuring < 500 µm^21^.

With recent technological advancement, there has been increasing interest in investigating the relationship between *in-vivo* corneal biomechanics and myopic progression. As SSI is evolved from an algorithm that apply numerical modelling input and output parameters CCT, bIOP, and the stiffness parameter at the highest concavity, aimed to provide a measure of tissue stiffness that is independent of both IOP and corneal geometry^10^, several studies reported significant differences in SSI and dynamic corneal responses among emmetropia, low myopia and high myopia groups^12,33–35^. Conversely, a study utilizing ORA did not discover changed biomechanical behaviours between the emmetropia and myopia^36^, which might be due to the low amount of the myopia. Discrepancies in these studies could be partially explained by different devices used that apply air puff differently to the cornea. It is critical to note that the Corvis ST produces a consistent air puff of 60 mmHg with each examination, whereas the ORA system adjusts the air puff pressure after the first applanation, thereby reducing the repeatability of subsequent measurements^7^. In our studies, it was noted that SSI was significantly different between the low and high myopia groups (Table 2), indicating that corneal physical properties may be progressively altered during myopia development.

Apart from the SSI, there are other Corvis ST derived corneal biomechanical parameters and indices that correspond to corneal stiffness such as Def A (2 mm), Def A ratio (2 mm), SP-A1, CBI and total biomechanical index (TBI)^37^^,^^38^. It has been shown in other studies that SP-A1, CBI and TBI biomechanical indices and deformation parameters are significantly correlated with myopia severity, with corneas in cases of high myopia being softer and easier to deform compared to those with mild/moderate myopia^39–41^. However, in the current study, no differences were seen between the 22–26.00 AL group and ≥ 26.00 AL groups in Def A (2 mm), Def A ratio (2 mm), CBI, SP-A1 or bIOP (Table 2). The variation between this study’s findings and that of others is likely due to differences in the inclusion and exclusion criteria, in particular the exclusion from this study of data from non-proportional eyes. It is plausible that the corneal dynamic parameters in response to an air-puff, although important for diagnosis for the corneal ectasia diseases and glaucoma, may not be sensitive to monitor myopia progression, since the mechanics of the axial myopia progression is mainly due to the elongation of posterior scleral and posterior vitreous body, vitreous depth, even though the mechanisms were not clearly demonstrated. Whereas the SSI, is a combined index of the cornea-sclera stiffness parameter, was associated with severity of myopia. This warrants further studies to see whether the Corvis ST corneal dynamic responses parameters could be affected by the myopia shifts or not.

The correlation between SER and SSI values shown in this study is comparable to that reported by Liu et al.^19^. In the study by Liu et al., values for SER in their ultra-high myopia group (− 11.59 ± 1.55) were significantly higher than those presented for the ≥ 26.00 AL group (− 9.45 ± 2.53) in this current study, and their values for SSI (0.771 ± 0.104) were significantly lower than reported here. In contrast, a study by Chu et al.^17^, which used a similar grouping to that used in this study, presented SSI values that were notably lower than those reported here. Differences in the value of SSI between the studies could be explained by the differing age ranges of their participants, since ocular stiffness is known to increase significantly with age^42^. For example, two studies involving younger children, reported no significant correlation between age and SSI in non-high and high myopia groups^17^, and no significant correlation between SSI and AL in severely elongated eyes^18^. Moreover, a large myopia cohort study in a Hong Kong population demonstrated that the cornea was more deformable in adults than in children^43^. Thus, SSI might exhibit differential trends between children and adults, and it may be more appropriate to separate the two populations when analysing altered ocular biomechanics in myopia. In the current study, to avoid the complication of data analysis from eyes that were not yet fully grown, only participants aged 18 years old or above were included.

Consistent with previous findings^10,18^, the current study also demonstrated a positive correlation between age and SSI (Table 3, Fig. 2A). However, studies that have focussed more extensively on the relationship between age and SSI have provided evidence that SSI increases significantly after the age of 35–40^11,44^. The small but nevertheless significant difference in the average age of the two groups in the current study (33.06 ± 10.34 years and 28.14 ± 8.74 years in the 22–26.00 AL group and ≥ 26.00 AL groups, respectively), may therefore be considered a limitation of the study.

While AL ≥ 26.00 mm is often used as the diagnostic threshold of high myopia in clinical studies^45^, it has been reported that the ratio AL/CR is better correlated with SER than AL alone and might therefore be a superior indicator for verifying myopia severity^17^^,^^20,46^. Chu et al. found that AL/CR was closely associated with SSI in a non-high myopia group and high myopia group^17^, but did not report refractive error in their study. Again, correlation results between the SSI and AL from others are conflicting. While Liu et al.^18^ found that the SSI was only negatively correlated with AL in the medium elongated eye group, but not in the severely elongated eye (AL ≥ 26 mm) group, Chu et al.^17^ found that there was no correlation between the AL and SSI in either the normal or the high myopia group. In the current study, SSI was only negatively correlated with biometric parameters including AL and AL/CR in the high myopia group. The difference between these findings may be due to the younger age range of participants in the previous studies and the inclusion of eyes with non-proportional AL/CR.

The AL/CR ratio was highly associated with myopia severity, as shown 3.22 ± 0.09 in our 22–26.00 AL group versus 3.51 ± 0.16 in our ≥ 26.00 AL group. In the COMET group study^20^, which involved 469 children aged between 6 and 12 years old, the mean amount of myopia increased from − 2.38 D at baseline to − 5.17 D at 14-years follow-up, while the average AL/CR ratio was 3.15 at baseline and increased to 3.31^20^. In the current ≥ 26.00 AL group, we also found that the AL/CR ratio showed the highest negative correlation with SSI compared to AL, SER and AL-ACD distance (Table 3), which is in accordance with the previous studies^47^.

Researchers have reported varying correlation between Corvis ST-derived parameters and corneal curvature^33,34^. Han et al. found that SSI was negatively correlated with the mean K value, but with a low correlation index of − 0.103^12^. However Liu et al. found that SSI had no correlation with Km and flat K^19^. A possible explanation for these observations is the diversified structural growth of eyes. Some eyes exhibit a flatter cornea along with a longer AL to compensate for neutralization of the refraction system. Furthermore, in a Shenzhen kindergarten population it was found that corneal power remained stable between 3 and 6 years of age, whereas AL, ACD, and AL/CR ratio increased, and lens power decreased^48^. Decreases in lens power, as opposed to corneal flattening, play the major role in the development of the refractive system in these early age groups^48^. In the COMET study^20^, there was a general trend for the cornea to flatten slightly with age over the 14-year study period. During the study years 6–14, there was very little additional flattening of the flattest meridian but the change in curvature was statistically significant (43.46 D to 43.38 D)^20^. Others also found that the structural cause of myopia in teenagers and adult-onset and adult-progression is vitreous chamber elongation, rather than the CR alteration^49,50–52^.

CCT is an important parameter in the diagnosis and management monitoring of ocular disease such as glaucoma and keratoconus, but its association with myopia remains elusive^53–55^. In our study, CCT was thicker in the high myopia group than the low myopia group. However, linear correlation between CCT and SER was very weak (r = − 0.253, *p* < 0.01) whereas Liu et al., found that the CCT had a continuous decreasing trend with AL^56^. Others found no difference in CCT between emmetropic and myopic eyes, and CCT did not correlate with the degree of myopia. It seems that the central cornea is not significantly involved in the process of myopic progression^57^. Consistent with this, the current study found a negative weak correlation between SSI and CCT in both groups, with only the > 26 AL group showing weak significance (Table 3). Longitude studies were warranted for further explanations on CCT changes with myopia shifts.

The current study has some limitations. Firstly, it did not include an emmetropia group. The rate of myopia is very high in China, and candidates participating in this study were enrolled from a laser centre where patients were awaiting refractive surgery. Secondly, the age, CCT and CR in the normal and ≥ 26.00 AL groups were different. However, a statistical adjustment was incorporated into the data analysis to balance the differences between the two groups. As discussed previously in relation to changes in SSI with age, the age range in this study was quite large (18—52-years old) and the number of enrolled participants aged over 40 was relatively high (40 cases in the 22–26.00 AL group, and 20 cases in the ≥ 26.00 AL group) to ensure age balance throughout the cohort. Thirdly, bIOP was used as a metric to estimate internal eye pressure, as demonstrated by ex-vivo experiments^58^. In the current study, the bIOP between the normal and ≥ 26.00 AL group was consistent after adjustment of age, CCT and CR, which increased the reliability of the SSI correlation analysis. Nevertheless, bIOP is only an approximation of the true intraocular pressure.

## Conclusions

In conclusion, the current results support the notion that corneal biomechanical changes accompany AL growth in the development of myopia. Corneal biomechanical indices such as SSI could be an indirect indicator of biomechanical behaviour, especially in severely myopic eyes. However, ideally longitudinal studies are needed to investigate the possibility that myopic eyes with lower stiffness may progress to high myopia and the usefulness of corneal biomechanical indices as predictors for myopia progression.

## Supplementary Information

Below is the link to the electronic supplementary material.

Supplementary Material 1

## Data Availability

Due to ethical restrictions, the clinical data supporting this study cannot be made openly available. De-identified data are available from the corresponding author upon reasonable request and with appropriate ethics approval.

## Abbreviations

ACD: Anterior chamber depth
AL: Axial length
AL/CR: Ratio of AL to radius (CR) of corneal curvature
AL-ACD: Axial length minus anterior chamber depth (ACD)
ANCOVA: Analysis of covariance
ARTh: Ambrósio Relational Thickness to the horizontal profile
bIOP: Biomechanically corrected intraocular pressure (IOP)
CBI: Corvis biomechanical index
CCT: Central corneal thickness
Corvis ST: Corneal Visualization Scheimpflug Technology
CR: Radius of corneal curvature
D: Dioptre
Def A ratio (2 mm): Ratio between the deflection amplitude at the apex and the deflection amplitude at 2 mm from the apex
Def A (2 mm): Deflection amplitude at 2 mm
IOP: Intraocular pressure
K: Keratometry
Mean K: Mean keratometry
SD: Standard deviation
SER: Spherical equivalent refraction
SP-A1: The stiffness parameter at the first applanation

## References

[1] Morgan, I. G. et al. The epidemics of myopia: Aetiology and prevention. *Prog. Retin. Eye Res.* **62**, 134–149 (2018). 28951126 10.1016/j.preteyeres.2017.09.004

[2] Holden, B. A. et al. Global prevalence of myopia and high myopia and temporal trends from 2000 through 2050. *Ophthalmology* **123**(5), 1036–1042 (2016). 26875007 10.1016/j.ophtha.2016.01.006

[3] Jonas, J. B., Ohno-Matsui, K. & Panda-Jonas, S. Myopia: Anatomic changes and consequences for its etiology. *Asia Pac. J. Ophthalmol. (Phila).* **8**(5), 355–359 (2019). 31425168 10.1097/01.APO.0000578944.25956.8b PMC6784857

[4] Dhakal, R., Vupparaboina, K. K. & Verkicharla, P. K. Anterior sclera undergoes thinning with increasing degree of myopia. *Invest. Ophthalmol. Vis. Sci.* **61**(4), 6 (2020). 32271887 10.1167/iovs.61.4.6 PMC7401898

[5] Boote, C. et al. Scleral structure and biomechanics. *Prog. Retin. Eye Res.* **74**, 100773 (2020). 31412277 10.1016/j.preteyeres.2019.100773 PMC7187923

[6] Meek, K. M. & Fullwood, N. J. Corneal and scleral collagens - a microscopist’s perspective. *Micron* **32**(3), 261–272 (2001). 11006506 10.1016/s0968-4328(00)00041-x

[7] Roberts, C. J. Concepts and misconceptions in corneal biomechanics. *J. Cataract Refract. Surg.* **40**(6), 862–869 (2014). 24857435 10.1016/j.jcrs.2014.04.019

[8] Ruberti, J. W., Sinha Roy, A. & Roberts, C. J. Corneal biomechanics and biomaterials. *Annu. Rev. Biomed. Eng.* **13**, 269–295 (2011). 21568714 10.1146/annurev-bioeng-070909-105243

[9] Ambrosio, R. J. R. I. et al. Dynamic ultra high speed Scheimpflug imaging for assessing corneal biomechanical properties. *Rev. Brasoftalmol.* **72**, 4 (2013).

[10] Eliasy, A. et al. Determination of corneal biomechanical behavior in-vivo for healthy eyes using CorVis ST tonometry: Stress-strain index. *Front. Bioeng. Biotechnol.* **7**, 105 (2019). 31157217 10.3389/fbioe.2019.00105 PMC6532432

[11] Liu, G. et al. Age distribution and associated factors of cornea biomechanical parameter stress-strain index in Chinese healthy population. *BMC Ophthalmol.* **20**(1), 436 (2020). 33143686 10.1186/s12886-020-01704-6 PMC7607623

[12] Han, F. et al. Effect of biomechanical properties on myopia: A study of new corneal biomechanical parameters. *BMC Ophthalmol.* **20**(1), 459 (2020). 33213408 10.1186/s12886-020-01729-x PMC7678063

[13] Padmanabhan, P., Lopes, B. T., Eliasy, A., Abass, A. & Elsheikh, A. In vivo biomechanical changes associated with keratoconus progression. *Curr. Eye Res.* **47**(7), 982–986 (2022). 35385372 10.1080/02713683.2022.2058020

[14] Nishida, T. et al. Evaluation of the relationship between the changes in the corneal biomechanical properties and changes in the anterior segment oct parameters following customized corneal cross-linking. *Clin. Ophthalmol.* **16**, 1909–1923 (2022). 35711971 10.2147/OPTH.S361836 PMC9192785

[15] G Y Liu LLJ, J Li, X L Du. 2022 Evaluation of two biomechanical stiffness indexes in the diagnosis of eartoconus and their changes after corneal collagen cross-linking surgery. Zhonghua Yan Ke Za Zhi. 58(8):8. 10.3760/cma.j.cn112142-20211027-00507 35959602

[16] Lu, N. J. H. F. et al. Effect of fluence levels on prophylactic corneal cross-linking for laser in situ keratomileusis and transepithelial photorefractive keratectomy. *Acta Ophthalmol.* **101**(2), 12 (2023). 10.1111/aos.15230 36794626

[17] Chu, Z. et al. The relationship between axial length/corneal radius of curvature ratio and stress-strain index in myopic eyeballs: Using Corvis ST tonometry. *Front. Bioeng. Biotechnol.* **10**, 939129 (2022). 36046672 10.3389/fbioe.2022.939129 PMC9420864

[18] Liu, G. et al. The effect of axial length elongation on corneal biomechanical property. *Front Bioeng. Biotechnol.* **9**, 777239 (2021). 34926423 10.3389/fbioe.2021.777239 PMC8677453

[19] Liu, Y., Pang, C., Ming, S. & Fan, Q. Effect of myopia and astigmatism deepening on the corneal biomechanical parameter stress-strain index in individuals of Chinese ethnicity. *Front Bioeng. Biotechnol.* **10**, 1018653 (2022). 36420440 10.3389/fbioe.2022.1018653 PMC9676639

[20] Scheiman, M. et al. Longitudinal changes in corneal curvature and its relationship to axial length in the Correction of Myopia Evaluation Trial (COMET) cohort. *J. Optom.* **9**(1), 13–21 (2016). 26564446 10.1016/j.optom.2015.10.003 PMC4705324

[21] Wang, X. et al. Assessment of corneal biomechanics, tonometry and pachymetry with the Corvis ST in myopia. *Sci. Rep.* **11**(1), 3041 (2021). 33542296 10.1038/s41598-020-80915-9 PMC7862660

[22] Bujang, M. A. & Baharum, N. Sample size guideline for correlation analysis. *World J. Soc. Sci. Res..* **3**, 37–46 (2016).

[23] Harper, A. R. & Summers, J. A. The dynamic sclera: Extracellular matrix remodeling in normal ocular growth and myopia development. *Exp. Eye Res.* **133**, 100–111 (2015). 25819458 10.1016/j.exer.2014.07.015 PMC4379420

[24] Boote, C. et al. Collagen organization in the chicken cornea and structural alterations in the retinopathy, globe enlarged (rge) phenotype–An X-ray diffraction study. *J. Struct. Biol.* **161**(1), 1–8 (2008). 17936639 10.1016/j.jsb.2007.08.015

[25] Morgan, S. R. et al. An x-ray scattering study into the structural basis of corneal refractive function in an avian model. *Biophys. J.* **104**(12), 2586–2594 (2013). 23790366 10.1016/j.bpj.2013.04.053 PMC3686341

[26] Kang, B. S., Lam, T. C., Cheung, J. K., Li, K. K. & Kee, C. S. Corneal proteome and differentially expressed corneal proteins in highly myopic chicks using a label-free SWATH-MS quantification approach. *Sci. Rep.* **11**(1), 5495 (2021). 33750851 10.1038/s41598-021-84904-4 PMC7943770

[27] Kang, B. S. et al. High myopia induced by form deprivation is associated with altered corneal biomechanical properties in chicks. *PLoS ONE* **13**(11), e0207189 (2018). 30419001 10.1371/journal.pone.0207189 PMC6231665

[28] Boote, C. et al. Ultrastructural changes in the retinopathy, globe enlarged (rge) chick cornea. *J. Struct. Biol.* **166**(2), 195–204 (2009). 19258040 10.1016/j.jsb.2009.01.009 PMC2680986

[29] Craig Boote, A. E. et al. The influence of lamellar orientation on corneal material behaviour: biomechanical and structural changes in an avian corneal disorder. *Invest. Ophthalmol. Vis. Sci.* **52**(3), 9 (2011). 10.1167/iovs.10-5962 PMC3101698 21051696

[30] Lopes, B. T. et al. Repeatability and reproducibility of intraocular pressure and dynamic corneal response parameters assessed by the Corvis ST. *J. Ophthalmol.* **2017**, 8515742 (2017). 28676837 10.1155/2017/8515742 PMC5476874

[31] Wang, W. et al. Corneal biomechanical metrics of healthy Chinese adults using Corvis ST. *Cont. Lens Anterior Eye.* **40**(2), 97–103 (2017). 27964894 10.1016/j.clae.2016.12.003

[32] Chen, X. et al. Reliability of corneal dynamic Scheimpflug analyser measurements in virgin and post-PRK eyes. *PLoS ONE* **9**(10), e109577 (2014). 25302580 10.1371/journal.pone.0109577 PMC4193795

[33] Yu, A. Y. et al. Corneal biomechanical properties in myopic eyes evaluated via Scheimpflug imaging. *BMC Ophthalmol.* **20**(1), 279 (2020). 32652982 10.1186/s12886-020-01530-w PMC7353814

[34] Sedaghat, M. R. et al. Corneal biomechanical properties in varying severities of myopia. *Front Bioeng Biotechnol.* **8**, 595330 (2020). 33553113 10.3389/fbioe.2020.595330 PMC7859342

[35] Long, W. et al. Characteristics of corneal biomechanics in chinese preschool children with different refractive status. *Cornea* **38**(11), 1395–1399 (2019). 31033694 10.1097/ICO.0000000000001971

[36] Biswas, S. & Biswas, P. Relationship between diurnal variation in intraocular pressure and central corneal power. *Optom. Vis. Sci.* **100**(1), 96–104 (2023). 36705719 10.1097/OPX.0000000000001974

[37] Vinciguerra, R. et al. Detection of keratoconus with a new biomechanical index. *J. Refract. Surg.* **32**(12), 803–810 (2016). 27930790 10.3928/1081597X-20160629-01

[38] Roberts, C. J. et al. Introduction of two novel stiffness parameters and interpretation of air puff-induced biomechanical deformation parameters with a dynamic Scheimpflug analyzer. *J. Refract. Surg.* **33**(4), 266–273 (2017). 28407167 10.3928/1081597X-20161221-03

[39] Li, D. L. et al. Refractive associations with corneal biomechanical properties among young adults: A population-based Corvis ST study. *Graefes Arch. Clin. Exp. Ophthalmol.* **262**(1), 121–132 (2024). 37401934 10.1007/s00417-023-06164-4

[40] El-Mayah, E., Albalkini, A. S. & Barrada, O. A. Characterization of corneal biomechanics using CORVIS ST device in different grades of myopia in a sample of middle eastern ethnicity. *Clin. Ophthalmol.* **18**, 901–912 (2024). 38529005 10.2147/OPTH.S451328 PMC10962271

[41] Shao, H. et al. Corneal biomechanical properties in myopic anisometropia measured by corneal visualization Scheimpflug technology. *Am. J. Transl. Res.* **17**(4), 2817–2825 (2025). 40385031 10.62347/EPRI9798 PMC12082562

[42] Elsheikh, A. et al. Assessment of corneal biomechanical properties and their variation with age. *Curr. Eye Res.* **32**(1), 11–19 (2007). 17364730 10.1080/02713680601077145

[43] Tang, S. M. et al. Association of corneal biomechanics properties with myopia in a child and a parent cohort: Hong Kong children eye study. *Diagnostics* **11**(12), 2357 (2021). 34943594 10.3390/diagnostics11122357 PMC8700309

[44] Kenia, V. P., Kenia, R. V., Pirdankar, O. H. & Bendre, P. Age-related variations in corneal stress-strain index in the Indian population. *Indian J. Ophthalmol.* **71**(6), 2421–2426 (2023). 37322652 10.4103/ijo.IJO_1980_22 PMC10418008

[45] Biswas, S. & Biswas, P. Longitudinal evaluation of the structural and functional changes associated with glaucoma in myopia. *Optom. Vis. Sci.* **97**(6), 448–456 (2020). 32511167 10.1097/OPX.0000000000001519

[46] Tao, Z. et al. A longitudinal study of the effect of ocular biometrics measures on myopia onset. *Graefes Arch. Clin. Exp. Ophthalmol.* **259**(4), 999–1008 (2021). 33201354 10.1007/s00417-020-05010-1

[47] Jong, M., Sankaridurg, P., Naduvilath, T. J., Li, W. & He, M. The relationship between progression in axial length/corneal radius of curvature ratio and spherical equivalent refractive error in myopia. *Optom. Vis. Sci.* **95**(10), 921–929 (2018). 30247237 10.1097/OPX.0000000000001281

[48] Guo, X. et al. Significant axial elongation with minimal change in refraction in 3- to 6-year-old Chinese preschoolers: The Shenzhen kindergarten eye study. *Ophthalmology* **124**(12), 1826–1838 (2017). 28711218 10.1016/j.ophtha.2017.05.030

[49] McBrien, N. A. & Adams, D. W. A longitudinal investigation of adult-onset and adult-progression of myopia in an occupational group. Refractive and biometric findings. *Invest. Ophthalmol. Vis. Sci.* **38**(2), 321–333 (1997). 9040464

[50] Jorge, J., Almeida, J. B. & Parafita, M. A. Refractive, biometric and topographic changes among Portuguese university science students: a 3-year longitudinal study. *Ophthalmic. Physiol. Opt.* **27**(3), 287–294 (2007). 17470242 10.1111/j.1475-1313.2007.00475.x

[51] Ruozhong Xie, X.-T.Z. et al. Correlation between myopia and major biometric parameters of the eye: A retrospective clinical study. *Optom. Vis. Sci.* **86**, 6 (2009). 10.1097/OPX.0b013e31819f9bc5 19349927

[52] Lee, J. T. L. et al. Progression and longitudinal biometric changes in highly myopic eyes. *Invest. Ophthalmol Vis Sci.* **61**(4), 34 (2020). 32334434 10.1167/iovs.61.4.34 PMC7401968

[53] Michael Mimouni, V. F. Yinon Shapira, Shmuel Graffi, Shmuel Levartovsky, Tzahi Sela, Gur Munzer, Igor Kaiserman Correlation between central corneal thickness and myopia. *Int. J. Ophthalmol.* **38**(6), 5 (2018). 10.1007/s10792-017-0766-1 29075941

[54] Chen, Y. C. K. T., Lee, H. J., Lee, S. H. & Lin, S. Y. Correlation between central corneal thickness and myopia in Taiwan. *Kaohsiung J. Med. Sci.* **30**(1), 5 (2014). 10.1016/j.kjms.2013.08.008 PMC11916882 24388054

[55] AlMahmoud, T. P. D., Munger, R. & Jackson, W. B. Correlation between refractive error, corneal power, and thickness in a large population with a wide range of ametropia. *Invest. Ophthalmol. Vis. Sci.* **52**(3), 8 (2011). 10.1167/iovs.10-5449 21051694

[56] Jin, G. et al. Corneal biometric features and their association with axial length in high myopia. *Am. J. Ophthalmol.* **238**, 45–51 (2022). 34896081 10.1016/j.ajo.2021.11.031

[57] Al-Mezaine, H. S. et al. The relationship between central corneal thickness and degree of myopia among Saudi adults. *Int. Ophthalmol.* **29**(5), 373–378 (2009). 18587538 10.1007/s10792-008-9249-8

[58] Eliasy, A. et al. Ex-vivo experimental validation of biomechanically-corrected intraocular pressure measurements on human eyes using the CorVis ST. *Exp. Eye. Res.* **175**, 98–102 (2018). 29908883 10.1016/j.exer.2018.06.013

