# Supplementary material for: The correlation between myopia severity and stress–strain index (SSI) using the Corneal Visualization Scheimpflug Technology (Corvis ST)
Source: Sci Rep. 2025 Nov 17;15:40103. doi: 10.1038/s41598-025-23834-x (PMC12623436; doi:10.1038/s41598-025-23834-x)
Supplement: Supplementary file 1 — Supplementary Material 1 [file 41598_2025_23834_MOESM1_ESM.pdf]

Supplementary Material

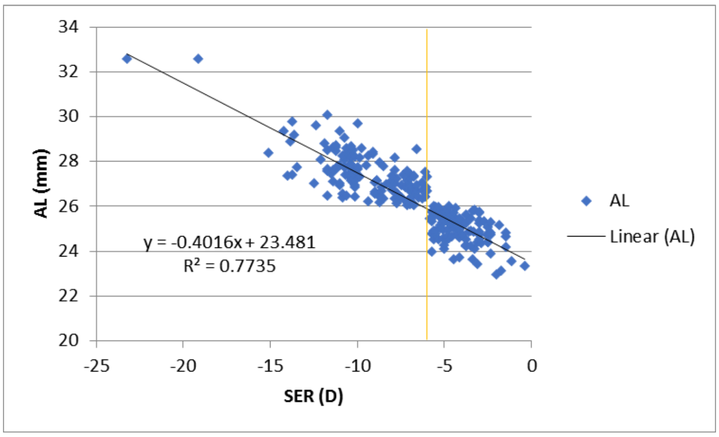

Figure S1. Correlation between SER and AL. The vertical yellow line marks the division between the two groups.

Table S1. Demographic and biometric data of the excluded 60 cases with non-proportional eye structure.

	22-26.00 AL group (43 OD eye) Mean±SD	≥26.00 AL group (17 OD eye) Mean±SD	P
**Sex**			<0.01†
	Female (76.7%)	Female (35.4%)	
	Male (23.3%)	Male (64.7%)	
**AL (mm)**	25.45±0.46	26.49±0.59	<0.01†
**Age (years)**	30.33±9.66	30.06±9.39	0.923
**SER (D)**	-7.49±1.32	-4.67±1.05	<0.01†
**CCT (μm)**	520.77±30.21	509.35±25.09	0.173
**ACD (mm)**	3.06±0.23	3.20±0.23	0.042*
**AL-ACD (mm)**	22.39±0.53	23.30±0.57	<0.01†
**CR (mm)**	7.54±0.19	8.06±0.33	<0.01†
**AL/CR ratio**	3.37±0.73	3.29±0.08	<0.01†

AL, axial length; SER, spherical equivalent refraction; CCT, corneal central thickness; ACD, anterior chamber depth; AL-ACD, axial length minus anterior chamber depth; CR, corneal radius value of the anterior surface around a 3mm ring; AL/CR ratio, axial length divided by corneal radius of the anterior surface around a 3mm ring.

**P-value <0.05; †P-value <0.01.*

for CCT, please change from 'corneal central thickness' to 'central corneal thickness'.

Table S2. Comparisons of Corvis ST derived DCRs and stiffness parameters between the 22-26.00 AL and ≥26.00 AL group of the non-proportional cases (60 cases).

	22-26.00 AL group<br>Mean±SD	≥26.00 AL group<br>Mean±SD	P
**bIOP (mmHg)**	14.84±2.19	15.14±2.14	0.635
**Def A（2mm）**	0.98±0.10	0.96±0.10	0.489
**DefA ratio (2mm)**	6.31±1.38	5.91±0.93	0.278
**CBI**	0.26±0.32	0.18±0.29	0.378
**SP-A1**	86.12±15.76	91.25±16.35	0.266
**SSI**	0.89±0.14	0.93±0.14	0.279

bIOP, biomechanically corrected intraocular pressure; Def A, deflection amplitude at 2mm; DefA ratio, deflection amplitude ratio at 2 mm; SP-A1, stiffness parameter at first applanation; SSI, stress-strain index. Independent t test was used in the comparisons of Corvis-ST derived DCRs and stiffness parameters between the 22.00 AL group and ⩾26.00 AL groups.

Table S3. Correlation analysis between SSI and biometric values for the excluded non-proportional eyes.

	22-26.00 AL group		≥26.00 AL group	
	r	*p*	*r*	*p*
**SSI vs AL**	-0.056	0.361	0.152	0.280
**SSI vs (AL-ACD)**	-0.051	0.372	0.217	0.202
**SSI vs SER**	0.318	0.019*	0.231	0.186
**SSI vs age**	0.023	0.443	0.348	0.086
**SSI vs bIOP**	0.553	<0.01†	0.602	<0.01†
**SSI vs AL/CR**	-0.477	<0.01†	-0.314	0.110
**SSI vs CR**	0.379	<0.01†	0.264	0.153

**P-value <0.05; †P-value <0.01; Pearson correlation was used in the correlations analysis.*

Table S4. Stepwise multivariate regression analysis of SSI and demographic and ocular values for the excluded non-proportional eyes.

parameters	*Age* (β *p*)	*AL* (β *p*)	SER (β *p*)	bIOP (β *p*)	CR (β *p*)	AL/CR (β *p*)	AL-ACD (β *p*)
**SSI in 22-26.00 AL group**	-	-	-	0.031, <0.01	-	-0.738, <0.01	-
**SSI in ≥26.00 AL group**	-	-	-	0.040, 0.01	-	-	-

By multiple stepwise regression, in the 22-26.00 AL, but higher than -6.00D SER group,
SSI = 2.927+0.031(bIOP)-0.738(AL/CR)

By multiple stepwise regression, in the >26.00 AL,
SSI = 0.003+0.040 (bIOP)
